# Editorial: Cross-disorder Genetics in Neuropsychiatry

**DOI:** 10.3389/fnins.2022.826300

**Published:** 2022-02-09

**Authors:** Evelien Van Assche, Eva C. Schulte, Ole A. Andreassen, Olav B. Smeland, Jurjen J. Luykx

**Affiliations:** ^1^Department of Psychiatry, University of Münster, Münster, Germany; ^2^Institute of Psychiatric Phenomics and Genomics (IPPG), University Hospital, University of Munich, Munich, Germany; ^3^Department of Psychiatry & Psychotherapy, University Hospital, University of Munich, Munich, Germany; ^4^Division of Mental Health and Addiction, NORMENT Centre, Oslo University Hospital, Institute of Clinical Medicine, University of Oslo, Oslo, Norway; ^5^KG Jebsen Centre of Neurodevelopmental Disorders, Institute of Clinical Medicine, University of Oslo, Oslo, Norway; ^6^Department of Psychiatry and Neuropsychology, School for Mental Health and Neuroscience, Maastricht University Medical Centre, Maastricht, Netherlands; ^7^Department of Psychiatry, UMC Utrecht Brain Center, University Medical Center Utrecht, Utrecht University, Utrecht, Netherlands; ^8^Outpatient Second Opinion Clinic, GGNet Mental Health, Warnsveld, Netherlands

**Keywords:** cross-disorder analyses, neuropsychiatric, genetics, genomics (G), neurology-clinical, psychiatry

Neurological and psychiatric disorders share a number of symptoms and signs. Although neurological diseases are often characterized by the presence of motor or sensory symptoms, these may also occur in psychiatric disorders: as part of the disorder or as comorbidity (Mittal et al., 2017). Behavioral or mood symptoms, on the other hand, are typically linked to psychiatric disorders but are often present as comorbid traits in neurological disorders as well. These observations fuel the concept of neuropsychiatry as a field in itself (Yudofsky and Hales, 2002). In contrast to the many shared characteristics between neurological and psychiatric disorders, recent findings point to a higher degree of genetic overlap between major psychiatric disorders in comparison to neurological disorders (Brainstorm Consortium et al., 2018; Olislagers et al., 2021). “Lumping and splitting” are common phenomena in medicine overall (Lüscher, 2019) and within psychiatry (Marquand et al., 2016). Some argue disciplines, diseases etc. should be seen as different entities (“splitting”), whereas others believe in continua without clear boundaries (“lumping”). This focus on the similarities and the differences between neurology and psychiatry illustrates the need for more fine-grained studies to elucidate to what degree neuropsychiatric features and symptoms, disorders and mechanisms overlap. This special issue invites readers to think critically about these concepts from a neuropsychiatric perspective and challenges the neurology-versus-psychiatry categorization at different levels. Although distinctions between both disciplines are often made, clinical presentations with both neurological and psychiatric symptoms are common. Huntington's disease (Ellis et al., 2020), neurodegenerative diseases in general, but also other disorders clearly challenge this notion of distinct and discernable nosologies. The aim of this special issue on “Cross-disorder Genetics in Neuropsychiatry” is to make readers familiar with the basics of the methods currently employed to study genetic overlap between neuropsychiatric disorders and some of the results these methods have yielded. We have placed a spotlight on studies that can increase our understanding of neurological, psychiatric, but foremost neuropsychiatric genetics. Given the advanced methodologies applied and the relevant clinical syndromes discussed, the papers in this issue are of interest to clinicians as well as researchers.

Most common human disorders are considered to have a polygenic architecture and their genetic interrelation is increasingly shown to be more complex than captured by genetic correlations (Torkamani et al., 2018; Smeland et al., 2019). Hence, focusing on specific disorders from the neuropsychiatry spectrum and overlapping genetic architectures can be a way forward. This is shown in the study by Monereo-Sanchez et al. presented in this issue, where overlapping genomic loci between major depressive disorder and dementia are identified, despite lacking evidence of a previous overall genetic correlation. The highly advanced methodology generated insight into the shared genetic architectures and the complexity of the genetic correlation of these disorders due to mixed directions of effects. Other studies have already shown shared genomic risk loci between neurological and psychiatric disorders, for example, in the case of Parkinson's disease and schizophrenia (Smeland et al., 2021).

Findings by Esposito et al., present an association between the Copy Number Variant (CNV) 16p11 deletion and cluster A personality disorder. This substantiates the concept that chromosomal rearrangements not only lead to neurological phenotypes, including epilepsy and intellectual disability and psychiatric phenotypes such as major psychosis but can also occur in pre-morbid personality traits. The 22q11 deletion is well known for its role in psychosis and schizophrenia (Kirov et al., 2015). The involvement of another CNV, 16p11 in particular, in paranoid personality traits is a novel insight with potential clinical impact, discussed in this issue. The authors argue that their observation could pave the way for the definition of new diagnostic subgroups of cluster A personality disorders and psychotic disorders and could influence the clinical management accordingly.

From a polygenic perspective, the cross-disorder concept typically focuses on a shared genetic vulnerability between different disorders. This holds true for disorders deemed “psychiatric” (Lee et al., 2019) and, possibly to a lesser extent, for “neurological” disorders (Brainstorm Consortium et al., 2018). The interest in the genetics' community in polygenic overlaps between disorders is on the rise (Grotzinger, 2021). Some go as far as to suggest a cross-disorder connectome network for all brain-related disorders (van den Heuvel and Sporns, 2019). From a single variant perspective, comorbid epilepsy can be a good indicator for an underlying monogenic condition in individuals with a predominantly “psychiatric” phenotype (Bassett et al., 2010). Highly relevant in this neuropsychiatric context, the role of large-effect, rare variation in epilepsy, is also addressed by two articles in this issue: Luo et al. highlight the homozygous mutation of the *VPS13A* gene in the context of Chorea-Acanthocytosis (ChAc) with epilepsy as the main presenting symptom, while Li et al. dissect the role of *CNNM2* variation in a phenotype characterized by intellectual disability and seizures but without hypomagnesemia.

As genetic data on neurological and psychiatric illnesses are expected to become larger and more detailed in the coming years, we anticipate that cross-disorder investigations such as those presented in this issue of Frontiers of Neuroscience present essential factors in disentangling the shared genetic underpinnings between neurological and psychiatric disorders and symptoms. Such cross-disciplinary efforts may facilitate the development of new prediction and treatment strategies for neuropsychiatric disorders and could significantly improve treatment of individuals suffering from a combination of neurological or psychiatric symptoms.

## Author Contributions

All authors listed have made a substantial, direct, and intellectual contribution to the work and approved it for publication.

## Conflict of Interest

The authors declare that the research was conducted in the absence of any commercial or financial relationships that could be construed as a potential conflict of interest.

## Publisher's Note

All claims expressed in this article are solely those of the authors and do not necessarily represent those of their affiliated organizations, or those of the publisher, the editors and the reviewers. Any product that may be evaluated in this article, or claim that may be made by its manufacturer, is not guaranteed or endorsed by the publisher.
